# The Illusion of Knowing in College: A Field Study of Students with a Teacher-Centered Educational Past

**DOI:** 10.5964/ejop.v15i4.1921

**Published:** 2019-12-19

**Authors:** Maura A. E. Pilotti, Khadija El Alaoui, Huda Mulhem, Halah A. Al Kuhayli

**Affiliations:** a College of Science and Human Studies, Prince Mohammad Bin Fahd University, Al Khobar, Kingdom of Saudi Arabia; Kingston University, London, UK

**Keywords:** self-assessment, performance, culture

## Abstract

In the present study, the tendency to overestimate performance (illusion of knowing) was examined in college students whose educational past experiences had emphasized verbatim learning. Female students enrolled in core curriculum classes were sampled. Classes taught by the same instructor were randomly assigned to a self-assessment practice condition, where students predicted their test and class performance and were asked to reflect on discrepancies between predictions and actual performance, or to a control condition. At the end of the semester, irrespective of condition, as performance declined on the final test, predictions of final test grades became more inflated, but less confident, indicating that students were aware of their own deficiencies. Overall, students in the practice condition displayed not only greater prediction accuracy, but also greater final test performance than students in the control condition. Practice, however, benefited the most self-assessment accuracy of students whose final test grades were just above the passing grade. Although the responses to self-assessment practice of students with a teacher-centered educational past were largely similar to the responses of students from Western countries reported in the extant literature, differences in impact and meaning could be inferred.

Metacognitive monitoring refers to activities that gather information about one’s own knowledge and skills in relation to the demands of a task (e.g., a test, a reading assignment, etc.), and then use it to adapt one’s actions to achieve desired performance standards (Koriat, Ackerman, Adiv, Lockl, & Schneider, 2014). According to this view of metacognition, two conditions must be met by learners in their pursuit of desired performance: First and foremost, learners must have a realistic grasp of their knowledge, understanding, and competence in relation to the demands of the situation (e.g., a test). Thus, the illusion of knowing phenomenon, which is a common judgment of overestimation of one’s knowledge, understanding, and/or competence is to be prevented at all costs (Dunning, Heath, & Suls, 2004; Glenberg, Wikinson, & Epstein, 1982). Second, learners must be willing and able to use strategically information about their knowledge, understanding, and competence to modulate thought and action during learning and testing (Avhustiuk, Pasichnyk, & Kalamazh, 2018).

This model of metacognition, according to which increasing students’ awareness of their knowledge in relation to the specific demands of a situation aids achievement, informs several activities that spontaneously take place in college classes around the globe. Consider, for instance, the instructors’ practice of reviewing the content of a test or assignment in class after having graded it, as well as students’ inquiries about their grades. The model has also generated targeted-interventions whose effects have yielded mixed results. An example is the practice of asking students to predict their exam scores before and after tests, which is later followed by a comparison of predicted and actual performance (Achacoso, 2004). This practice is firmly rooted in the belief that inaccurate judgments about knowledge and capabilities are likely to interfere with students’ recognition of their deficiencies and needs for improvement (Dunning, Johnson, Ehrlinger, & Kruger, 2003). As a result, it relies on a set of sequential assumptions: First, students who experience the illusion of knowing will likely overestimate their performance and feel confident about their overestimations, only to discover afterward that they were wrong. Second, discrepancies between predicted and actual performance will make students aware of what they do and do not know. Third, such awareness will lead them to make needed adjustments to their learning. It is reasonable to ask whether these assumptions are supported by unequivocal facts.

Evidence exists that predictions of test performance may improve students’ accuracy of self-assessment (Dunlosky, Serra, Matvey, & Rawson, 2005), but not always performance (Miller & Geraci, 2011a). In addition, students’ accuracy of self-assessment decreases as their performance declines (Jee, Wiley, & Griffin, 2006; Pallier et al., 2002). Namely, the most vulnerable students in the class are those that overestimate their performance the most (Bol & Hacker, 2001; Hacker, Bol, Horgan, & Rakow, 2000; Kruger & Dunning, 1999). However, Miller and Geraci (2011b), who differentiated functional overconfidence (e.g., predicting a grade higher than the one received on a test), from subjective overconfidence (e.g., being certain that one’s prediction is correct), found that vulnerable students place little confidence in their inflated estimates (Miller & Geraci, 2011b). Thus, low-performing students are not “blissfully incompetent” (Williams, 2004), “unskilled” and “unaware” (Ehrlinger, Johnson, Banner, Dunning, & Kruger, 2008), as it has been claimed, but rather their overestimates appear to be a form of wishful thinking that temporarily shields their self-concept from adverse events. Yet, evidence mostly comes from the Western world.

The cultural environments in which people have been raised may shape learning in different ways, thereby promoting some abilities over others (Hofstede, 1986; Lundeberg, Fox, Brown, & Elbedour, 2000). For instance, a culture that relies on oral transmission of knowledge is likely to promote verbatim learning in its youngsters, and thus enhance their verbatim memory skills relative to those of young Westerners (Rogoff, 1990). In such a culture, older adults are often given the key role of transmitting knowledge, including cultural traditions and history. Not surprisingly, a teacher-centered pedagogy (Stipek, 1991; Weimer, 2002), which emphasizes verbatim learning and sees the teacher as the authority ultimately responsible for assessment, comes to shape formal education. In a nutshell, this is the history of primary and secondary education in the Kingdom of Saudi Arabia (Al Khazim, 2003; Al Lily, 2011; Al Mezani, 2010; Günther, 2006; Hamdan, 2014; Mahrous & Ahmed, 2010). Although verbatim learning is largely inadequate for successful performance in college, it is a habit that is resistant to change. Students revert to it as they find it a comforting escape from the more nebulous problem-solving approach of higher education. Through the lenses of this form of teacher-centered education, there is no uncertainty regarding what constitutes successful performance (Oettingen, 1995; Weimer, 2002). Performance attainment is defined by students’ ability to repeat the words uttered by the instructor or written in textbooks (Säljö, 1981). The principal source of assessment is the instructor who is regarded as “the sage on the stage.” Obviously, when faced with learning tasks of hundreds of pages, students cannot rely entirely on a reproductive approach to learning (Biggs, 1976), which is their preferred mode. Nevertheless, they resist fully exploring a more reflective and active approach, including organizing and linking new to known materials (Säljö, 1981). For instance, students may mention selected facts, and describe discrete contents, but rarely venture into reorganizing contents or attempt to go beyond the information given (Entwistle, 1979). Not surprisingly, disaster strikes when this habit of information processing is applied to prepare for tests that require not just retention and local comprehension of key concepts, but also application, analysis, evaluation, and generation of original work (Bloom, 1976). Even then, students may resist abandoning their preferred mode of processing entirely (Fareh, 2010; McLellan, 2012). The habit of faithful reproduction is also selectively reinforced in particular classes, such as Arabic and Islamic Studies (Douglass, & Shaikh, 2004) and Oral Communication, where verbatim learning is often seen as an efficient (i.e., quick and reliable) strategy of information acquisition and retention.

## Rationale of the Present Field Study

The present study is based on the metacognitive model described above, according to which self-regulation of thought and action for the purpose of optimizing learning or testing is dependent on students’ ability to gather realistic information about the knowledge and skills they possess. Thus, to induce metacognitive awareness (Flavell, 1979) of what is known and what is not known, students are asked to predict their test and class performance (functional confidence) and report their degree of trust in the predictions made (subjective confidence) at four different times during the semester. They are also required to examine their predictions against the grades they actually receive and reflect on any discrepancies.

Students with a teacher-centered educational past offer an uncharted ground for testing the power of recurrent and explicit self-assessment on metacognitive awareness and performance as they habitually rely on the instructor for self-assessment. It is hypothesized that in such students self-assessment practice, relative to a condition without practice, will improve metacognitive awareness and thus estimates of final test performance made at the end of the semester. Based on the findings of Miller and Geraci (2011b), students with less than desirable performance are expected to benefit the most from self-assessment practice in terms of improved metacognitive awareness rather than actual performance. However, such students in KSA may also be those that most firmly rely on verbatim learning and on the assessment provided by the instructor (i.e., the expert). Thus, students who routinely treat the instructor as the principal source of appraisal may discount the feedback provided by explicit self-assessment, rendering self-assessment practice moot. Namely, a pedagogical tool, which has been proven to be effective in the Western world, may not be equally effective in the Middle East (Mahrous & Ahmed, 2010; Smith, 2006). Alternatively, its novelty may grab students’ attention, leading them to recognize the usefulness of the information it provides. As a new gadget whose utility is suddenly recognized due to compulsory use, self-assessment practice may induce a change not only in metacognitive awareness, but also in performance through new habit formation. Additionally, students may be more or less sensitive to an intervention intended to enhance metacognitive awareness depending on the adverse experiences they encountered in college. Consider that time constraints, poor academic background, and limited course-relevant experiences and skills have been reported to be key sources of withdrawal from courses (Hayward, 2003; Lee & Choi, 2011; Poling, 1979). Thus, the number of college classes students completed compared to those they attempted (i.e., class completion ratio) can be treated as an index of experiences in which performance did not match expectations. Studies have shown that, in addition to interventions that encourage or require students to use support services (Gordanier, Hauk, & Sankaran, 2018), simply reminding students of their standing in a course can improve performance and thus reduce course withdrawal rates (Chen & Okediji, 2014; Smith, White, Kuzyk, & Tierney, 2018). It is reasonable to expect responses to a technique that is intended to promote self-understanding, such as self-assessment practice, to vary with students’ class completion rate. Namely, we predict that smaller class completion ratios may be associated with students’ increased sensitivity to overt self-assessment practice, as such practice in class both encourages and normalizes the task of understanding one’s current situation.

If functional and subjective confidence ratings are considered local judgments, it is reasonable to ask whether they are informed by broader traits, such as general self-efficacy. General self-efficacy refers to students’ underlying confidence to perform well across diverse tasks and situations. It is considered a motivational trait (Chen, Gully, Whiteman, & Kilcullen, 2000) positively correlated with task completion rates (Eden, 1984, 1988; Pajares, 1996), persistence (Bandura, 1977; Pajares, 1997), motivation and engagement (Bandura, 1989; Bandura & Schunk, 1981). In the Western world, some evidence exists that it can predict functional confidence (Avhustiuk et al., 2018), but evidence of its ability to predict academic achievement is mixed (Pintrich, Smith, Garcia, & McKeachie, 1993; Wilhite, 1990; Zeegers, 2004). In students with a teacher-centered educational past, general self-efficacy may predict functional and subjective confidence ratings if such local judgments are the reflection of a motivation to change established learning habits. It may even predict performance if motivation has led to action. In addition, general self-efficacy may specifically modulate students’ responses to self-assessment practice so that students with varying confidence in their abilities are more or less sensitive to the practice of estimating grades.

## Method

### Participants

The participants were 685 female college students at a University in the Eastern Province of KSA. Participants reported Arabic as their first language and English as their second language. For university admission, students had demonstrated English language competence through standardized English proficiency tests. Students completed primary and secondary schooling in KSA. They were all commuters from nearby towns or cities who were enrolled in a Core Curriculum class, which offered practice in basic academic skills (e.g., writing, speaking, reasoning, etc.) across a variety of topics or focused on a specific domain of knowledge, such as history, Arabic culture and religion, and psychology. If a student was enrolled in more than one of the selected Core Curriculum classes, withdrew, or missed the midterm or the final test, her data were not included in the current sample (12.51%). Weimer’s dimensions differentiating educational approaches (Weimer, 2002) were used to classify students as possessing a teacher-centered educational past and habits of information acquisition reliant on verbatim learning. Due to gender-segregation rules, a corresponding male sample was unattainable.

### Materials and Procedure

Convenience sampling (i.e., consent of the instructor, and minimal or absent overlap of students) was used to select 32 classes. For each instructor, sections of the same class were randomly assigned to either a self-assessment practice condition (*n* = 337 students) or a control condition (*n* = 348 students) to ensure that instructors would have half of the classes assigned to practice and the other half to control. Instructors were either local hires or foreigners, but all possessed graduate degrees obtained outside KSA.

In Table 1, the practice condition involved 4 phases in which students estimated their class grade (phase 1 and phase 3) as well as their grade on the midterm (phase 2) and final exams (phase 4), before and after each test. As per institutional guidelines, all classes required a midterm test and a final test, which 1) included a fair mixture of short-answer and multiple-choice questions, and 2) assessed not only retention and understanding of key information, but also use of knowledge, such as application, analysis, and evaluation (see Bloom’s taxonomy; Bloom, 1976).

**Table 1:** Procedure of the Field Experiment and Descriptive Statistics (Means and Standard Errors of the Mean) by Phase and Condition

Variable	Practice Condition	Control Condition
Phase 1		
Estimation of Class Grades		
Accuracy	2.39 (0.50)	—
Confidence	2.50 (0.05)	—
Phase 2		
Midterm Test Grades	84.19 (0.85)	83.17 (0.84)
Estimation of Midterm Test Grades		
Prospective Accuracy	3.84 (0.87)	—
Prospective Confidence	2.11 (0.05)	—
Retrospective Accuracy	0.83 (0.77)	—
Retrospective Confidence	1.96 (0.06)	—
Phase 3		
Estimation of Class Grades		
Accuracy	1.85 (0.43)	—
Confidence	2.10 (0.06)	—
Phase 4		
Final Test Grades	86.75 (0.77)	82.56 (0.76)
Estimation of Final Test Grades		
Prospective Accuracy	2.33 (0.70)	4.69 (0.69)
Prospective Confidence	2.23 (0.06)	2.28 (0.06)
Retrospective Accuracy	0.92 (0.68)	3.57 (0.67)
Retrospective Confidence	2.13 (0.06)	2.28 (0.06)
End of Semester		
Class Grades	89.06 (0.52)	88.17 (0.51)

Phase 1 was initiated during the first 2 weeks of the semester. Students assigned to the practice condition were given a sheet on which they were to predict their class grade on a scale from 0 to 100, as well as to express their confidence in the prediction made on a scale from 0 (not at all confident) to 4 (extremely confident). All students, irrespective of the condition to which they were assigned, completed the New General Self-Efficacy (NGSE; Chen, Gully, & Eden, 2001) inventory. The NGSE required students to report the extent to which they agreed with each of eight statements of general confidence in one’s abilities on a scale from strongly disagree (1) to strongly agree (5).

Phase 2 of the practice condition was implemented during the administration of midterm tests. Before starting the midterm test and after having completed it, each student predicted her performance. For both prospective and retrospective estimates, the student expressed her confidence in the prediction made. Instructors discussed the content of the midterm test upon returning it to students to ensure adequate performance feedback. Phase 3 of the practice condition was administered during the last 2 weeks of the semester. Students were again asked to predict their class grade as well as express their subjective confidence in the prediction made. Estimates and confidence ratings of phases 1–3, which were made on separate sheets of paper, were returned to students within a week so that information was available for review before additional predictions were required. Students were encouraged to reflect on discrepancies between predictions and actual performance, and, where needed, to consider remedies either by themselves or with the aid of the instructor. They were informed that explicit and periodic assessment of their performance followed by self-reflection would offer them the opportunity to understand what they acquired and how they acquired it in reference to the demands posed either by a specific activity or by the collection of activities that a class placed on them. As predictions of class grades could be verified only at the end of the semester, students were encouraged to check the running average of all completed activities on their course management system to evaluate the extent to which their predictions matched their actual performance. Phase 4 was implemented during the administration of the final tests. Each student predicted her performance on the final test, as well as expressed her confidence in the prediction made before and after the test. To minimize the temptation of aspirational predictions, in all phases, students were reminded to report what they reasonably believed to be their grade at the time of the inquiry. They were explicitly discouraged to report what they wished their grade to be.

As the semester moved closer to its end, all students were expected to have increased their knowledge of the demands of the class in which they were enrolled (i.e., as assessed by differences between phase 1 and phase 3), and of the more specific demands of its tests (i.e., as assessed by differences between phase 2 and phase 4). Thus, the control condition, where students did not predict their grades in phases 1–3, served to offer a “business-as-usual” setting, against which the effects of self-assessment practice could be measured. As in the practice condition, however, instructors discussed the content of the midterm test upon returning it to students to ensure adequate performance feedback (see Mahrous & Ahmed, 2010). Furthermore, students assigned to the control condition predicted their grades and reported their confidence in their predictions during phase 4 so that functional and subjective confidence estimates expressed without any practice could be compared with those made in the condition where estimates were a recursive exercise.

## Results

All results reported in this section are considered significant at the .05 level. The first set of analyses involved comparisons between control and practice conditions to assess initial baseline equivalence. Thus, at the start of the semester, the grade-point average (GPA) values of the students assigned to the control condition (*M* = 3.10, *SEM* = 0.03) and practice condition (*M* = 3.09, *SEM* = 0.03) were compared. They were found not to be different, *F* < 1, *ns.* Because GPA represents a global criterion, subject to influences from a large array of factors, completion rate (i.e., number of courses completed over number of courses attempted) as well as general self-efficacy (Bandura, 1977), a motivational factor that might impact performance, were also examined. Self-efficacy, measured by the New General Self-Efficacy inventory (NGSE; Chen et al., 2001), did not differ between control (*M* = 4.30, *SEM* = 0.02) and practice (*M* = 4.36, *SEM* = 0.02), *F*(1, 683) = 3.31, *p* = .069. Similarly, class completion rates were not different between control (*M* = 0.95, *SEM* = 0.01) and practice (*M* = 0.94, *SEM* = 0.01), *F*(1, 683) = 1.99, *p* = .159.

In compliance with the guidelines of the Office for Human Research Protections of the U.S. Department of Health & Human Services, data collected were entered into a database in which each student was assigned a random number. Thus, upon completion of the study, records were devoid of identifying information. The data analyzed involved those of students for whom the midterm, final test, class grades, and functional and subjective confidence ratings were available. Prediction accuracy (i.e., functional confidence) was measured as a difference between estimated grades and actual test or class grades. Thus, a value of 0 represented perfectly accurate self-assessment, a value greater than 0 indicated an overestimation and a value below 0 represented an underestimation.

### Predictions of Final Test Grades: Students’ Accuracy of Estimation and Confidence

**Figure 1:**
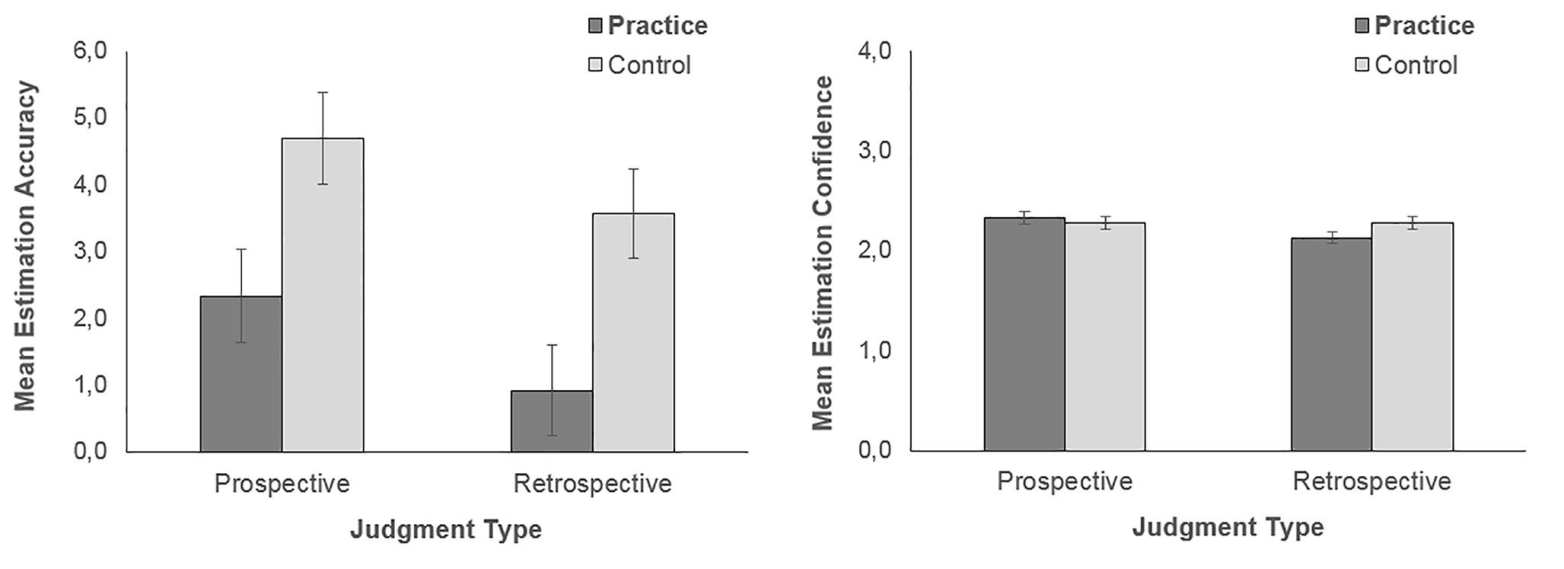
Mean prospective and retrospective prediction accuracy (left) and subjective confidence (right) of final test grades as a function of condition. Standard errors of the mean are represented by the error bars attached to each column.

At the end of the semester, predictions made by students who practiced self-assessment were expected to be more accurate and confident than those of students assigned to the control condition. Performance might follow suit if self-assessment practice affected students’ study activities. However, because knowledge of a test increased after having taken it, all students were expected to be more accurate and confident in their predictions after having completed the final test. To test these predictions, a 2 timing of prediction (prospective and retrospective) X 2 condition (control and practice) mixed factorial ANOVA was conducted on functional confidence (i.e., accuracy) and subjective confidence of estimation of final test grades. Descriptive statistics are displayed in Table 1. Retrospective estimations were more accurate than prospective estimations, *F*(1, 683) = 33.64, *MSE* = 16.36, *p* < .001, $\eta_{p}^{2}$ = .047. As illustrated in Figure 1, estimations in the practice condition were superior to those of the control condition, *F*(1, 683) = 7.04, *MSE* = 304.45, *p* = .008, $\eta_{p}^{2}$ = .010 (interaction: *F* < 1, *ns*). Subjective confidence in estimated values, however, was not sensitive to either condition or timing of prediction (*F*s ≤ 2.34, *p ≥* .127).

**Figure 2:**
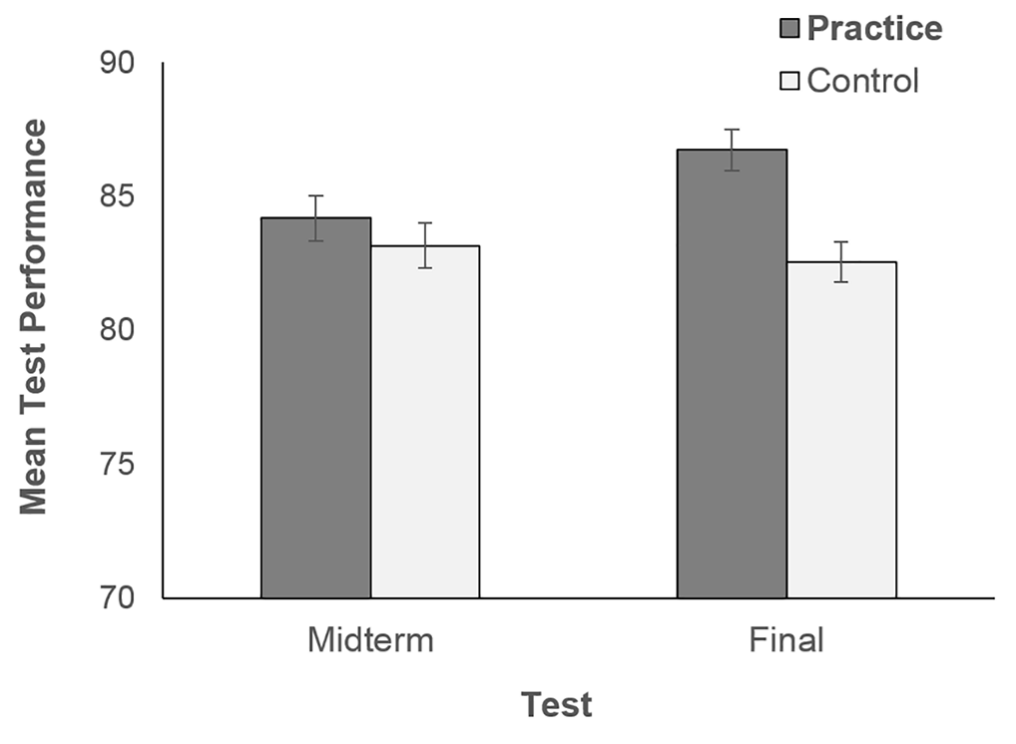
Midterm and final grades as a function of condition. Standard errors of the mean are represented by the error bars attached to each column.

A 2 condition (control and practice) X 2 test (midterm and final exam) mixed factorial ANOVA on students’ grades indicated that not only prediction accuracy, but also overall performance was higher in the practice than in the control condition, *F*(1, 683) = 6.65, *MSE* = 348.96, *p* = .001, $\eta_{p}^{2}$ = .010. No significant differences between tests were observed, *F*(1, 683) = 3.01, *p =* .069. Yet, a significant interaction suggested a more complex pattern, *F*(1, 683) = 8.81, *MSE* = 98.24, *p* = .003, η_p_*^2^ =* .013 (see Figure 2). Tests of simple effects indicated that students’ final test performance was higher in the practice than in the control condition, *t*(683) = 3.87, *p* < .001. If predictions of final grades reflected the impact of self-assessment practice on students’ test performance, the absence of a difference between conditions in midterm test grades (*t* < 1*, ns*) indicated that explicit self-assessment required time before it could be beneficial to performance.

### Predictions of Class Grades: Students’ Accuracy of Estimation and Confidence

Predictions of class grades (see Table 1) were considered more complex than those involving test grades as they would require students to combine estimates of the outcomes of an array of activities carried out during an entire semester. Nevertheless, as students’ knowledge of class demands improved from the beginning (phase 1) to the end of the semester (phase 3), predictions in the practice condition were expected to be more accurate and confident**.** A one-way ANOVA indicated that accuracy did not change, *F*(1, 336) = 2.43, *p* = .120*,* from phase 1 to phase 3. However, subjective confidence declined, *F*(1, 336) = 42.15, *MSE* = .63, *p* < .001, $\eta_{p}^{2}$ = .111, suggesting that increasing knowledge of class demands made students less likely to trust their estimates. Consistent with the null finding regarding prediction accuracy, a one-way ANOVA on class grades indicated that performance in the practice and control condition did not differ, *F*(1, 683) = 1.49, *p =* .223.

In our study, class grades not only combined performance evaluations from multiple sources (e.g., tests and assignments), but also received less clearly targeted feedback (Mahrous & Ahmed, 2010), thereby being more difficult to estimate than final test grades. According to students, predictions of final test grades could benefit from the objective feedback obtained from the midterm, whereas predictions of class grades on phase 3 had to rely in part on the running average of all completed activities on the course management system. Running averages were considered by students less informative than the feedback gathered from estimating midterm performance and completing the midterm. The nebulous feedback of class grades appeared only to have made students more cautious. Indeed, as self-assessment practice increased from phase 1 to phase 3, it had no visible impact on the accuracy of estimation of class grades or on actual grades, but it promoted less confidence in the estimates made.

### Prediction Accuracy and Subjective Confidence as a Function of Performance Level and Condition

Regression analyses were conducted to assess whether high and low performing students’ functional and subjective confidence would be differentially sensitive to self-assessment practice (see Table 2). In these analyses, condition (control and practice), performance (final test grades) and the interaction term served as predictors. Performance, which was treated as a continuous variable, was centered on its mean value (Field, 2009). Retrospective ratings of final test grades and related confidence served as the outcome variables because they provided a stringent test of the impact of self-assessment practice above and beyond the knowledge of class and test demands that would be normally acquired by students at the end of the semester.

**Table 2:** Regression Analyses of Retrospective Prediction Accuracy and Subjective Confidence of Final Test Grades as a Function of Condition, Performance, and Interaction

Predictor	*B*	*SE*	Beta	*t*	*p*
Prediction Accuracy					
Constant	+1.956	0.421			
Condition	+0.283	0.599	+0.011	+0.47	.636
Performance*	−0.700	0.021	−0.801	−32.87	<.001
Interaction*	+0.080	0.021	+0.090	+3.74	<.001
Subjective Confidence					
Constant	+2.345	0.058			
Condition*	−0.260	0.082	−0.117	−3.18	.002
Performance*	+0.025	0.003	+0.319	+8.51	<.001
Interaction*	−0.006	0.003	−0.076	−2.04	.042

*Note.* Prediction Accuracy (Functional Confidence): *R* = 0.79. Subjective Confidence: *R* = 0.32.

*Significant contribution of predictor to dependent variable.

Performance contributed to the accuracy of retrospective predictions (see top half of Table 2), indicating that predictions changed from liberal (i.e., overestimation) to conservative (i.e., underestimation) as performance increased. The significant interaction, however, revealed that estimation accuracy benefited from practice selectively, depending on the students’ performance level. The same regression analysis performed on students’ subjective confidence regarding their retrospective predictions uncovered a significant independent contribution of condition and performance (see bottom half of Table 2), indicating that self-assessment practice made students less confident in their predictions and that confidence decreased as performance declined. Yet, the significant interaction revealed that confidence declines as a function of assessment practice were dependent upon students’ performance level. Regression analyses pertaining to prospective predictions and related subjective confidence are reported in the Appendix 1. They showed a similar pattern for prospective estimates, and an attenuated pattern for subjective confidence, whereby confidence and performance merely decreased together.

The contribution of performance to both outcome variables illustrated a phenomenon also reported by Miller and Geraci (2011). Namely, as performance declined, retrospective estimates became more inflated, but less confident. To better understand the patterns yielded by the interaction of condition and performance, we organized students into four performance levels based on their final test grades: (highest: 100–90%; high: 89–80%; low: 79–67%; and lowest: below 67%, which corresponded to failing the test). Table 3, which reports descriptive statistics, uncovers several patterns involving the selective impact of self-assessment practice. The practice exercise benefited the most the accuracy of retrospective estimates of students with barely passing (i.e., low) performance, whereas it reduced the subjective confidence in the estimates made as performance increased.

**Table 3:** Mean Retrospective Prediction Accuracy and Subjective Confidence of Final Test Grades, Grouped by Performance Level and Condition

Performance Level (%)	Practice Condition	Control Condition	Difference
Accuracy			
Lowest	+ 24.23 (1.15)	+24.53 (1.30)	−0.03
Low	+3.83 (1.45)	+12.67 (0.84)	−8.84
High	−0.53 (1.03)	−1.81 (0.72)	−1.28
Highest	−4.33 (0.55)	−4.91 (0.75)	−0.58
Subjective Confidence			
Lowest	1.63 (0.16)	1.42 (0.17)	+0.21
Low	1.62 (0.20)	2.02 (0.11)	−0.40
High	1.93 (0.14)	2.38 (0.10)	−0.45
Highest	2.36 (0.07)	2.67 (0.10)	−0.31

*Note.* Standard errors of the mean are in parentheses.

### Prediction Accuracy, Subjective Confidence, and Performance as a Function of Self-Efficacy, Completion Rates, and Condition

Self-assessment of test performance, including functional and subjective confidence, might be the expression of students’ broader sense of trust in their abilities (as indexed by general self-efficacy), and failed expectations (as indexed by class completion rates). Regression analyses were conducted to determine the contribution of these factors, in addition to self-assessment practice, to functional and subjective confidence and final test grades. As discussed earlier, retrospective ratings of final test grades and related confidence served as the outcome variables because they provided a stringent test of the impact of self-assessment practice above and beyond the knowledge of class and test demands that students would normally acquire in a semester. In these analyses, condition (control and practice), general self-efficacy, class completion rates and two key interaction terms (condition X general self-efficacy, and condition X class-completion rates) served as predictors. Interaction terms were selected in recognition of the fact that the impact of self-assessment practice might vary depending on students’ class completion rates or self-efficacy. Class completion rates and general self-efficacy, which were treated as continuous variables, were centered on their respective mean values (Field, 2009).

The results of these regression analyses are displayed in Table 4. The only significant contributor to functional and subjective confidence ratings was self-assessment practice. However, both self-assessment practice and general self-efficacy (NGSE) contributed to students’ final test performance. Specifically, students’ self-efficacy increased with their test performance, suggesting that a broader sense of confidence in one’s ability may promote a more efficient execution of test requirements. Yet, it did not predict local judgments, such as functional and subjective confidence of final test grades, suggesting that such judgments may be considered the expression of a motivational state rather than of a motivational trait.

**Table 4:** Regression Analyses of Retrospective Prediction Accuracy and Subjective Confidence of Final Test Grades, and Final Test Grades as a Function of Condition, General Self Efficacy (GSE), Class Completion Rates (CCR), and Relevant Interactions

Predictor	*B*	*SE*	Beta	*t*	*p*
Prediction Accuracy					
Constant	+3.574	0.672			
Condition*	−2.573	0.957	−0.103	−2.69	.007
GSE	−1.928	1.202	−0.061	−1.60	.109
CCR	−2.882	4.660	−0.024	−0.62	.536
Interaction (GSE & Condition)	−0.272	1.202	−0.009	−0.23	.821
Interaction (CCR & condition)	+6.640	4.660	+0.056	+1.43	.155
Subjective Confidence					
Constant	+2.286	0.060			
Condition*	−0.171	0.085	−0.077	−2.00	.046
GSE	+0.196	0.107	−0.070	+1.83	.068
CCR	−0.239	0.415	−0.023	−0.57	.566
Interaction (GSE & Condition)	+0.085	0.107	+0.030	+0.79	.428
Interaction (CCR & condition)	−0.025	0.415	−0.002	−0.06	.952
Final Test Performance					
Constant	+82.638	0.759			
Condition*	+3.916	1.082	+0.137	+3.62	<.001
GSE*	4.516	1.359	+0.125	+3.32	<.000
CCR	−2.316	5.266	−0.017	−0.44	.660
Interaction (GSE & Condition)	+0.662	1.359	+0.018	−0.49	.626
Interaction (CCR & condition)	−6.263	5.266	−0.046	−1.19	.235

*Note.* Prediction Accuracy (Functional Confidence): *R* = 0.14. Subjective Confidence: *R* = 0.10. Final Test Performance: *R* = 0.20.

*Significant contribution of predictor to outcome variable.

### Qualitative Evidence

Informal focus groups (as part of debriefing sessions) and observations were conducted at the end of the semester with students and instructors regarding the impact of self-assessment practice. Given the hectic nature of end-of-the-semester activities, convenience sampling of students and instructors was utilized. To ensure candor and respect anonymity, individual comments were recorded without a link to the self-assessment data collected in class. These inquiries led to the following additional qualitative findings: 1) Practice helped students keep attention focused on discrepancies between predicted grades and actual grades. It also made them acutely aware of the distinction between desired grades and grades that could be predicted if exerted effort, adopted study strategies, and demands of tests or class are carefully and objectively considered. 2) Attention to self-assessment helped some students feel more in control of their learning and led them to seek a better understanding of how their study efforts and strategies to prepare for tests could be related to their performance. (3) Noticeably diminished were instructors’ reports of grade groveling inside and outside the classroom, grade appeals, as well as reports of adversarial interactions between instructors and students regarding performance. Of course, given our reliance on convenience sampling, and the absence of a link between individual comments and the data collected in class, these opinions and observations are informative, but of limited impact and in need of additional testing.

## Discussion

The overall findings of the present investigation can be summarized in three points: First, at the end of the semester, practice with self-assessment improved students’ ability to predict final test performance (i.e., functional confidence) and their actual performance, but did not markedly affect their subjective confidence in the predictions made. Second, practice with self-assessment did not improve students’ ability to predict class grades from the start (phase 1) to the end of the semester (phase 3), but it made subjective confidence in their predictions more conservative. Third, general self-efficacy along with self-assessment practice predicted final test performance, but not functional or subjective confidence.

These findings obscured two key individual differences. First, at the lowest performance level, overestimations dominated predictions, whereas at the highest level, underestimations prevailed. Yet, the students whose prediction accuracy of final test performance benefited the most from practice were those with score just above the passing level (68–79%). As performance increased, students responded to self-assessment practice by becoming less confident in their estimates. These patterns indicated that self-assessment practice effects were selective, either targeting deficiencies in metacognitive awareness in students with barely passing grades or restraining subjective confidence in students who already possessed adequate metacognitive awareness and performance. Second, low performers overestimated their final test grades, but exhibited little confidence in their optimistic estimations. High performers underestimated their final test grades but did so with greater confidence. Thus, patterns of underestimation and overestimation associated with performance levels did not appear to be merely statistical artifacts (see Krueger, & Mueller, 2002) or undiluted differences in metacognitive skills (Kruger & Dunning, 1999).

They appeared to be sensible judgments based on students’ knowledge of their capabilities in relation to the demands of the situation they faced. As judgments are made within a socio-cultural context, they are also likely to reflect self-presentation tactics. It has been argued that a person’s confidence is shaped by the culture in which he/she lives (Hofstede, 1986). Unlike Western culture, which is, in essence, individualistic, Arabic culture is collectivistic/situational, whereby the needs and goals of the group to which one is affiliated take precedence over the needs and goals of the individual (Feghali, 1997). Thus, if Western students see test and class performance as having implications primarily for themselves, Arab students see performance as having implications for themselves within the social fabric of the group(s) to which they belong. Lundeberg et al. (2000) reported that students of Arab descent (Palestinian) tended to be overconfident compared to Western students. Although we found the self-assessment patterns of students of Arab descent (KSA nationals) to be largely equivalent to those of Western students, differences may exist in the meaning attributed to overestimations. Indeed, for Arab students, overestimations may be the byproducts of strategies intended to maintain positive views not only of themselves in the communities to which they belong, but also of such communities. For Western students, overestimations may be the byproducts of self-serving strategies intended to maintain a positive self-image. Thus, the impact of self-assessment practice on Arab students with a teacher-centered training can be considered broader, implicating key aspects of the social fabric in which students carve their identities.

A reproductive approach, which is often promoted by a teacher-centered education, and a reflective/active approach generally lead students to focus on different kinds of information when learning. These approaches have consequences, affecting what students learn, and ultimately their grades (Säljö, 1981). Although it is generally believed that a reflective/active approach has a positive impact on learning outcomes, the examination of the relationship between learning approaches and academic outcomes (e.g., GPA) has produced inconsistent results (Heikkilä, Niemivirta, Nieminen, & Lonka, 2011; Watkins & Hattie, 1981; Zeegers, 2001, 2004; Zeegers & Martin, 2001). Qualitative evidence from students with a teacher-centered educational past, such as ours, may help understand these inconsistencies in the literature. In fact, for students with a teacher-centered educational past, not only the reflective/active approach is adopted on a limited basis when circumstances demand it, but also a reproductive approach may be seen as effective under other circumstances. For instance, consider test questions that require definitions of key concepts to be applied to real-life situations, analyzed, or evaluated (Bloom, 1976). According to our debriefing and focus-group data, students see memorization and comprehension of definitions as a means to ensure retrieval of key concepts during a test that may ask them to explain, compare, contrast, or apply such concepts to real-life situations. Thus, awareness of one’s performance, induced by repeated exercise of grade estimation, may not be sufficient to quickly induce new habit formation in students with a teacher-centered educational past (i.e., rejection of their proclivities for replication). Rather it may encourage a more optimal calibration of learning approaches based on the demands of the variety of situations that students face at different times.

If self-assessment practice can shape metacognitive awareness, functional and subjective confidence may be expected to be related to a motivational trait, such as self-efficacy. In the present study, self-efficacy predicted final test grades, but not self-assessment accuracy and confidence. Thus, our findings are consistent with those illustrating a positive correlation between self-efficacy and academic achievement (Pintrich et al., 1993; Zeegers, 2004). However, they are inconsistent with those of Avhustiuk et al. (2018), who found that students’ general self-efficacy increased with the accuracy of self-assessment. In an earlier study (Al Kuhayli et al., 2019), involving a larger group of students with a teacher-centered educational past, we found no link between general self-efficacy and measures of self-assessment and performance. These inconsistencies require further examination.

Aspects of the methodology of the present field study may raise questions about the unambiguous interpretation of our findings, such as random assignment of sections of the same class, rather than participants, to conditions. We relied for data collection on courses, but we selected instructors who taught at least two different sections of the same course so that for each section assigned to control another was assigned to practice. As per information collected from debriefings of students after the study was completed, the estimates made in the practice condition tended to be perceived by the students as routine class activities, perhaps less noteworthy than the content of assignments and tests, but as required as class attendance, timing submission of work, etc. Students mentioned their heavy workload, usually long commutes, and family obligations as their primary concerns. Main topics of conversation outside the classroom were reported to be specific troublesome assignments, instructors’ demands and behavioral styles, and social life matters. Thus, it is not surprising that students assigned to the practice condition did not appear to treat the estimation task as a significant topic of discussion outside of the classroom. Lastly, although our sample was female-only, it is important to note that cross-cultural evidence of gender differences in functional and subjective confidence is meager. Namely, contrary to stereotypes, women appear to be just as confident as men (Lundeberg et al., 2000).

## Appendix

**Appendix 1:** Regression Analyses of Prospective Prediction Accuracy and Subjective Confidence of Final Test Grades as a Function of Condition, Performance, and Interaction.

Predictor	*B*	*SE*	Beta	*t*	*p*
Prediction Accuracy					
Constant	+2.993	0.403			
Condition	+0.783	0.573	+0.030	+1.37	.172
Performance*	−0.750	0.020	−0.834	−36.79	<.001
Interaction*	+0.071	0.020	+0.078	+3.50	.001
Subjective Confidence					
Constant	+2.332	0.054			
Condition	−0.142	0.076	−0.069	−1.86	.064
Performance*	+0.021	0.003	+0.296	+7.82	<.001
Interaction	−0.003	0.003	−0.044	−1.17	.243

*Note.* Prediction Accuracy (Functional Confidence): *R* = 0.82. Subjective Confidence: *R* = 0.29.

*Significant contribution of predictor to outcome variable.
